# Supplementary material for: Comparative genomics provides new insights into the diversity, physiology, and sexuality of the only industrially exploited tremellomycete: Phaffia rhodozyma
Source: BMC Genomics. 2016 Nov 9;17:901. doi: 10.1186/s12864-016-3244-7 (PMC5103461; doi:10.1186/s12864-016-3244-7)
Supplement: Additional file 6: — List of orphan genes with links to PFAM (related to Additional file 1: Table S1). (ZIP 1428 kb) [file 12864_2016_3244_MOESM6_ESM.zip › BLAST_HTML_FTR/G05803_P.html]

BLAST Search Results


```
BLASTP 2.2.27+


Reference:
Stephen F. Altschul, Thomas L. Madden, Alejandro A. Schäffer,
Jinghui Zhang, Zheng Zhang, Webb Miller, and David J. Lipman (1997),
"Gapped BLAST and PSI-BLAST: a new generation of protein database
search programs", Nucleic Acids Res. 25:3389-3402.


Reference for
composition-based statistics:
Alejandro A. Schäffer, L. Aravind, Thomas L. Madden, Sergei
Shavirin, John L. Spouge, Yuri I. Wolf, Eugene V. Koonin, and
Stephen F. Altschul (2001), "Improving the accuracy of PSI-BLAST
protein database searches with composition-based statistics and
other refinements", Nucleic Acids Res. 29:2994-3005.


Database: nr
           71,551,133 sequences; 26,053,659,533 total letters


Query= G05803_P

Length=188
                                                                      Score     E
Sequences producing significant alignments:                          (Bits)  Value

emb|CDZ97469.1|  hypothetical protein [Xanthophyllomyces dendrorh...   392    1e-136
emb|CDZ97459.1|  hypothetical protein [Xanthophyllomyces dendrorh...   299    7e-100
emb|CDZ96930.1|  hypothetical protein [Xanthophyllomyces dendrorh...   209    5e-64 
emb|CDZ96635.1|  hypothetical protein [Xanthophyllomyces dendrorh...   201    1e-60 
ref|WP_038671947.1|  ribonuclease G [Pelosinus sp. UFO1] >gb|AIF5...  38.5    3.1   
ref|XP_003054191.1|  predicted protein [Nectria haematococca mpVI...  37.4    7.6   


 >emb|CDZ97469.1| hypothetical protein [Xanthophyllomyces dendrorhous]
Length=187

 Score =  392 bits (1006),  Expect = 1e-136, Method: Compositional matrix adjust.
 Identities = 187/187 (100%), Positives = 187/187 (100%), Gaps = 0/187 (0%)

Query  1    MPEDKYPNRRYKPDTLDEIYTPVREPFVCDVYCDCWLITATQGGQLGDFPLSELVAQSSG  60
            MPEDKYPNRRYKPDTLDEIYTPVREPFVCDVYCDCWLITATQGGQLGDFPLSELVAQSSG
Sbjct  1    MPEDKYPNRRYKPDTLDEIYTPVREPFVCDVYCDCWLITATQGGQLGDFPLSELVAQSSG  60

Query  61   RAELRELQPSEFWKAAAAHGINTDNLVYRVSNMKLYVGTRDGETHWNLDEAMYKMGRNAE  120
            RAELRELQPSEFWKAAAAHGINTDNLVYRVSNMKLYVGTRDGETHWNLDEAMYKMGRNAE
Sbjct  61   RAELRELQPSEFWKAAAAHGINTDNLVYRVSNMKLYVGTRDGETHWNLDEAMYKMGRNAE  120

Query  121  HHPLMDYIVIPEDPVSSGKLVTSMDEDASIVVSNILAEGHSKALRLDESTEDQSVGEEGE  180
            HHPLMDYIVIPEDPVSSGKLVTSMDEDASIVVSNILAEGHSKALRLDESTEDQSVGEEGE
Sbjct  121  HHPLMDYIVIPEDPVSSGKLVTSMDEDASIVVSNILAEGHSKALRLDESTEDQSVGEEGE  180

Query  181  AETKAKL  187
            AETKAKL
Sbjct  181  AETKAKL  187


>emb|CDZ97459.1| hypothetical protein [Xanthophyllomyces dendrorhous]
Length=201

 Score =  299 bits (765),  Expect = 7e-100, Method: Compositional matrix adjust.
 Identities = 146/146 (100%), Positives = 146/146 (100%), Gaps = 0/146 (0%)

Query  42   QGGQLGDFPLSELVAQSSGRAELRELQPSEFWKAAAAHGINTDNLVYRVSNMKLYVGTRD  101
            QGGQLGDFPLSELVAQSSGRAELRELQPSEFWKAAAAHGINTDNLVYRVSNMKLYVGTRD
Sbjct  56   QGGQLGDFPLSELVAQSSGRAELRELQPSEFWKAAAAHGINTDNLVYRVSNMKLYVGTRD  115

Query  102  GETHWNLDEAMYKMGRNAEHHPLMDYIVIPEDPVSSGKLVTSMDEDASIVVSNILAEGHS  161
            GETHWNLDEAMYKMGRNAEHHPLMDYIVIPEDPVSSGKLVTSMDEDASIVVSNILAEGHS
Sbjct  116  GETHWNLDEAMYKMGRNAEHHPLMDYIVIPEDPVSSGKLVTSMDEDASIVVSNILAEGHS  175

Query  162  KALRLDESTEDQSVGEEGEAETKAKL  187
            KALRLDESTEDQSVGEEGEAETKAKL
Sbjct  176  KALRLDESTEDQSVGEEGEAETKAKL  201


>emb|CDZ96930.1| hypothetical protein [Xanthophyllomyces dendrorhous]
Length=234

 Score =  209 bits (531),  Expect = 5e-64, Method: Compositional matrix adjust.
 Identities = 109/183 (60%), Positives = 126/183 (69%), Gaps = 0/183 (0%)

Query  1    MPEDKYPNRRYKPDTLDEIYTPVREPFVCDVYCDCWLITATQGGQLGDFPLSELVAQSSG  60
            MP+ +YPNR+YKPD  DEIY       VCD  C+C L+ A+         L+ELVA SSG
Sbjct  23   MPKVEYPNRKYKPDYDDEIYIRPGRKIVCDDNCECSLMHASALDGGWGVTLNELVALSSG  82

Query  61   RAELRELQPSEFWKAAAAHGINTDNLVYRVSNMKLYVGTRDGETHWNLDEAMYKMGRNAE  120
            RAELRELQP  FW   AAHG+NTD+LVY V++MKLYVG    ET W L  AM  MG NAE
Sbjct  83   RAELRELQPRGFWNVVAAHGVNTDDLVYSVTDMKLYVGAHVMETQWKLFSAMSDMGNNAE  142

Query  121  HHPLMDYIVIPEDPVSSGKLVTSMDEDASIVVSNILAEGHSKALRLDESTEDQSVGEEGE  180
            HHPLMDYIV+     SSG  VT M +DASIVVSNILAEG+SK L+LDE+TED  V E  E
Sbjct  143  HHPLMDYIVVRSSTASSGIPVTRMHDDASIVVSNILAEGYSKVLQLDETTEDLIVDEAAE  202

Query  181  AET  183
             +T
Sbjct  203  GDT  205


>emb|CDZ96635.1| hypothetical protein [Xanthophyllomyces dendrorhous]
Length=268

 Score =  201 bits (512),  Expect = 1e-60, Method: Compositional matrix adjust.
 Identities = 106/188 (56%), Positives = 132/188 (70%), Gaps = 5/188 (3%)

Query  5    KYPNRRYKPDTLDEIYTPVREPFVCDVYCDCWLITATQG----GQLG-DFPLSELVAQSS  59
            ++PNRRYKP   D IY    + F CD  CDC L+   +G    G +G +F LS  V+ SS
Sbjct  81   EFPNRRYKPGYNDWIYVRPGKEFACDDNCDCSLLHGWKGCEAWGGMGREFSLSNFVSLSS  140

Query  60   GRAELRELQPSEFWKAAAAHGINTDNLVYRVSNMKLYVGTRDGETHWNLDEAMYKMGRNA  119
            GRAELRELQPSEFW+A  A+ ++ D+LVYRVS+MKLYVG R+  T   L  AM  MG N 
Sbjct  141  GRAELRELQPSEFWEAVGAYDVDADDLVYRVSDMKLYVGGREVRTQLGLSRAMELMGDNE  200

Query  120  EHHPLMDYIVIPEDPVSSGKLVTSMDEDASIVVSNILAEGHSKALRLDESTEDQSVGEEG  179
            EH PLMDYIV+PED V SG  VTSM +DA++VVSNILAEG+SK L+LDE+TED  V E  
Sbjct  201  EHQPLMDYIVVPEDTVFSGTPVTSMYDDATVVVSNILAEGYSKVLQLDETTEDLIVDEAA  260

Query  180  EAETKAKL  187
            E +T +++
Sbjct  261  EGDTVSQV  268


>ref|WP_038671947.1| ribonuclease G [Pelosinus sp. UFO1]
 gb|AIF52475.1| ribonuclease, Rne/Rng family [Pelosinus sp. UFO1]
Length=480

 Score = 38.5 bits (88),  Expect = 3.1, Method: Compositional matrix adjust.
 Identities = 31/98 (32%), Positives = 50/98 (51%), Gaps = 12/98 (12%)

Query  78   AHGINTDNLVYR----VSNMKLYVGTRDGETHWNLDEAMYKMGRNAEHHPLMDYIVIPED  133
            A+G   D L Y     VS ++LY G  D  TH+ LDE + K+G+ +       YIVI + 
Sbjct  228  AYGRVCDLLKYASPELVSRVRLYQGKEDIFTHYGLDEELNKLGQRSVWLKCGGYIVIDKT  287

Query  134  P------VSSGKLVTSMDEDASIVVSNILAEGHSKALR  165
                   V++GK V S++   ++  +N+  E  S+ +R
Sbjct  288  EALTVIDVNTGKFVGSINLSDTVFQTNL--EAASEIVR  323


>ref|XP_003054191.1| predicted protein [Nectria haematococca mpVI 77-13-4]
 gb|EEU48478.1| predicted protein [Nectria haematococca mpVI 77-13-4]
Length=701

 Score = 37.4 bits (85),  Expect = 7.6, Method: Compositional matrix adjust.
 Identities = 28/97 (29%), Positives = 48/97 (49%), Gaps = 7/97 (7%)

Query  87   VYRVSNMKLYVGTRDGETHWNL--DEAMYK-----MGRNAEHHPLMDYIVIPEDPVSSGK  139
            V  +SN++ +  T D E  W+L  D A+++     +   AE H  M    +P DP+ + +
Sbjct  304  VTDLSNVQEFYQTDDDEGGWDLRPDWAVWRKDDKVLYVAAEKHGRMVLWKLPSDPLKARE  363

Query  140  LVTSMDEDASIVVSNILAEGHSKALRLDESTEDQSVG  176
            L   + ED S+V +++L +G S  +      E  S G
Sbjct  364  LPEPIHEDGSVVEAHLLGDGDSLLITSKSRIESSSYG  400


Lambda      K        H        a         alpha
   0.316    0.134    0.403    0.792     4.96 

Gapped
Lambda      K        H        a         alpha    sigma
   0.267   0.0410    0.140     1.90     42.6     43.6 

Effective search space used: 750352639660


  Database: nr
    Posted date:  Sep 23, 2015 12:05 AM
  Number of letters in database: 26,053,659,533
  Number of sequences in database:  71,551,133


Matrix: BLOSUM62
Gap Penalties: Existence: 11, Extension: 1
Neighboring words threshold: 11
Window for multiple hits: 40
```
